# Supplementary material for: Daphnia magna egg piRNA cluster expression profiles change as mothers age
Source: BMC Genomics. 2022 Jun 8;23:429. doi: 10.1186/s12864-022-08660-z (PMC9175491; doi:10.1186/s12864-022-08660-z)
Supplement: Supplementary file 1 — Additional file 1: Figure S1. Clustered heatmaps for differentially expressed piRNA clusters with a TPM > 1000 in egg replicates across F0, egg and F1 generations. [file 12864_2022_8660_MOESM1_ESM.pdf]

Figure S1. Clustered heatmaps for differentially expressed piRNA clusters with a TPM > 1000 in egg replicates across F0, egg and F1 generations.

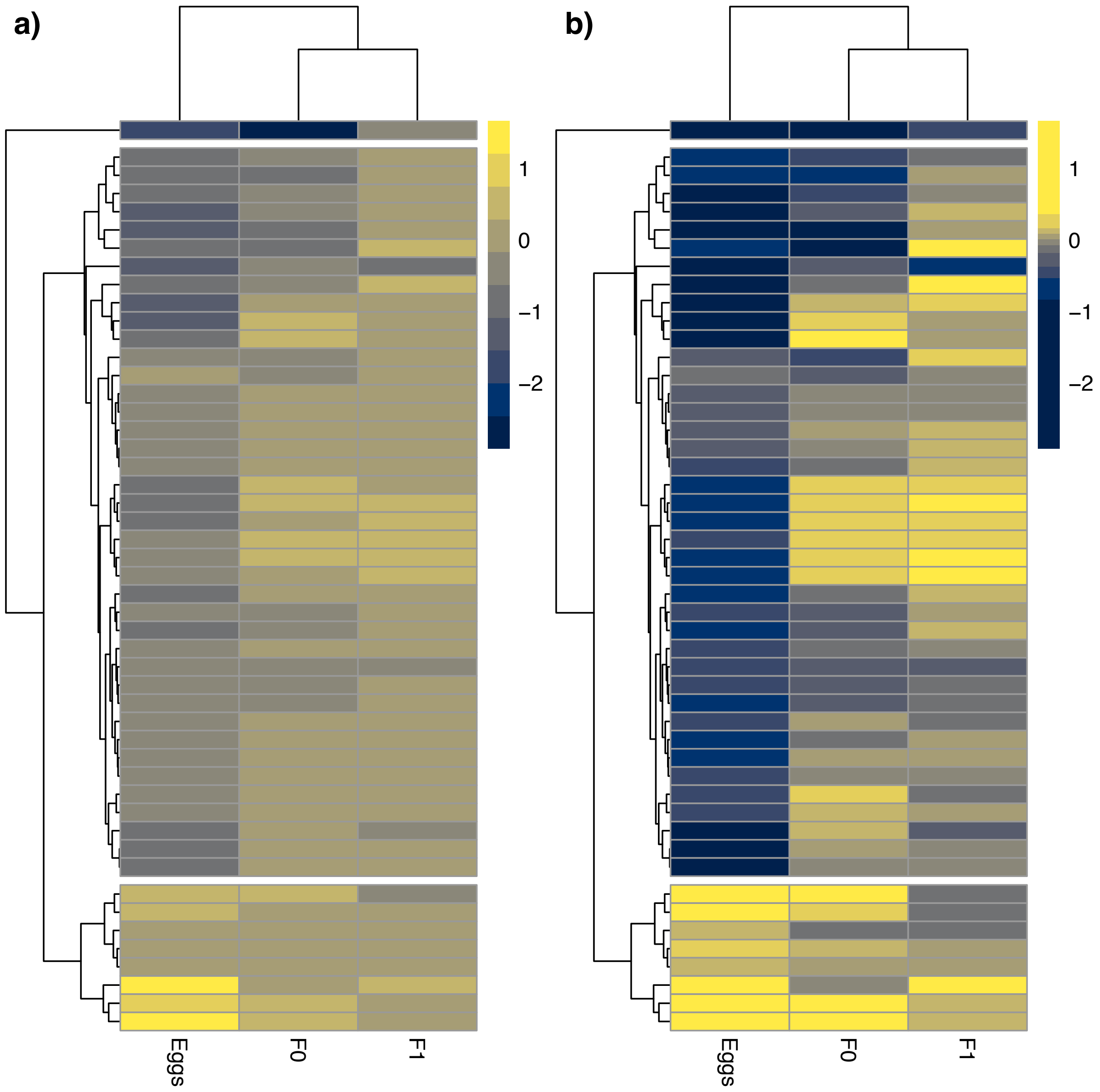

Clusters are shaded by log_2-fold change: negative values shaded in blue indicate piRNA clusters more highly expressed in 1st clutches; positive values in yellow indicate piRNA clusters more highly expressed in 5th. Part a) DE clusters shaded by log2-fold change alone. Part b) clusters shaded by log2-fold change split into 10 quantiles of equal size, this indicates directionality better than part a) due to modest log2-fold of piRNA cluster changes across the experiment. PiRNA Clusters were split into three groups by the three deepest splits in the hierarchy.
